# Preventing the Progression of Prehypertension to Hypertension: Role of Antihypertensives

**DOI:** 10.1007/s11906-014-0505-1

**Published:** 2014-11-29

**Authors:** Flávio Danni Fuchs, Renato Bandeira de Mello, Sandra Costa Fuchs

**Affiliations:** 1 Division of Cardiology, Hospital de Clínicas de Porto Alegre, UFRGS, Ramiro Barcelos 2350, Sala 2060, 90035-903 Porto Alegre, Brazil; 2 Division of Internal Medicine, Hospital de Clínicas de Porto Alegre, UFRGS, Porto Alegre, Brazil; 3 Graduate Program in Cardiology, School of Medicine, UFRGS, Porto Alegre, Brazil

**Keywords:** Hypertension, Prehypertension, Prevention, Antihypertensive drugs

## Abstract

Recent guidelines for the diagnosis and management of hypertension reversed the historical trend to recommend lower blood pressure (BP) thresholds to diagnose hypertension in high-risk individuals, such as patients with diabetes and elderly patients. The decision to raise the BP thresholds for diagnosis of hypertension in patients with diabetes was mostly based on the findings of the ACCORD trial. Nonetheless, the results of the ACCORD trial are within the predicted benefit to prevent coronary artery disease and stroke by meta-analysis of randomized controlled trials (RCT), particularly in regard to the prevention of stroke. The Eighth Joint National Committee (JNC 8) did not address prehypertension. There are many RCT done in individuals with prehypertension and concomitant cardiovascular disease showing the benefit of treatment of these patients. Trials exploring the efficacy of interventions to prevent cardiovascular disease in individuals with prehypertension free of cardiovascular disease would be hardly feasible in face of the low absolute risk of these individuals. Considering the risks of prehypertension for cardiovascular disease and the fast progression to hypertension of a large proportion of individuals with prehypertension, it is worth to consider drug treatment for individuals with prehypertension. RCT showed that the progression to hypertension can be partially halted by BP-lowering agents. These and ongoing clinical trials are herein revised. Prehypertension may be a window of opportunity to prevent hypertension and its cardiovascular consequences.

## Introduction

The concept of prehypertension was proposed by the Seventh Joint National Committee (JNC 7) [1] report and generated controversy and new venues for research but was not addressed in the Eighth Joint National Committee (JNC 8) report [2•]. The European guidelines maintained the definitions of high-normal blood pressure (BP) [3] but did not recommend the prescription of BP-lowering drugs at this stage. We believe that that there are sound evidences to diagnose prehypertension and to investigate the consequences and measures of control of prehypertension, including the use of antihypertensives in selected patients.

## New Thresholds for the Diagnosis of Hypertension in the US and European Hypertension Guidelines

International guidelines for the diagnosis and management of hypertension were recently released [2•, 3]. A historical trend to recommend lower BP thresholds to diagnose hypertension in high-risk individuals was unexpectedly reversed. BP targets for the treatment were modified accordingly. Therefore, individuals who had hypertension before are now normotensives. In the USA, the proportion of older adults (≥60 years) with treatment-eligible hypertension decreased from 68.9 % (95 % confidence interval (CI), 66.9–70.8 %) under JNC 7 [3] to 61.2 % (95 % CI, 59.3–63.0 %) under the 2014 BP guideline [4•].

European guidelines established 140/85 mmHg as the new target to diagnose hypertension in patients with diabetes [3], instead of 130/80 mmHg recommended by the previous guidelines [5]. In addition, the current guideline recommends that the drug treatment should be started at BP equal or higher than 160 mmHg in elderly patients. The JNC 8 report [2•] presents a radical shifting from the JNC 7 [1] in several aspects. In regard to diagnostic thresholds and goals of treatment, the JNC 8 report establishes higher diagnostic cutoff values for BP in some conditions as well. For individuals older than 60 years, the report established 150 mmHg of systolic BP as the new diagnostic limit and the target of treatment, keeping 90 mmHg for diastolic BP. For patients with diabetes and chronic kidney disease, the current recommendations are the same as those for adults without diabetes (140/90 mmHg), in comparison with 130/80 mmHg recommended in the JNC 7 report. The JNC 8 report did not address prehypertension, a condition proposed in the JNC 7 and that is a current focus of research, both in terms of risk for cardiovascular disease and therapeutic approach.

The authors of the US and the European guidelines based their new target recommendations for BP treatment on the results of randomized clinical trials (RCT). They should be complimented by the initiative, which recognizes the primacy of the results of RCT to justify medical decisions. Nonetheless, the results of the ACCORD trial could have another interpretation [6•]. Moreover, they left aside the results of many trials that were done with patients with normal BP and cardiovascular disease, assuming that these trials were not applicable to patients with hypertension. These and other issues related to goals of treatment and the J-shaped phenomenon were recently revised [7]. In our view, the diagnostic thresholds for the diagnosis of hypertension should not be raised, but should be lowered. Thresholds to diagnose hypertension in the upcoming years may be those currently recommended for the diagnosis of prehypertension.

## What the ACCORD Trial Really Shows

The ACCORD study tested the hypothesis that lowering blood pressure beyond guideline recommendations would confer higher cardiovascular protection [6•]. The trial assessed the incidence of cardiovascular events in patients with diabetes assigned to intensive therapy, targeting systolic pressure below 120 mmHg, in comparison with the standard therapy, targeting systolic pressure of less than 140 mmHg. The incidence of coronary heart disease events—the primary endpoint—was not statistically different between treatment arms, and serious adverse events were three times more frequent in the intensive arm. Based on these findings, guidelines recommended 140/90 mmHg as the goal in the management of hypertension for patients with diabetes. Nonetheless, the findings of the ACCORD trial may have a different interpretation.

**Fig. 1:**
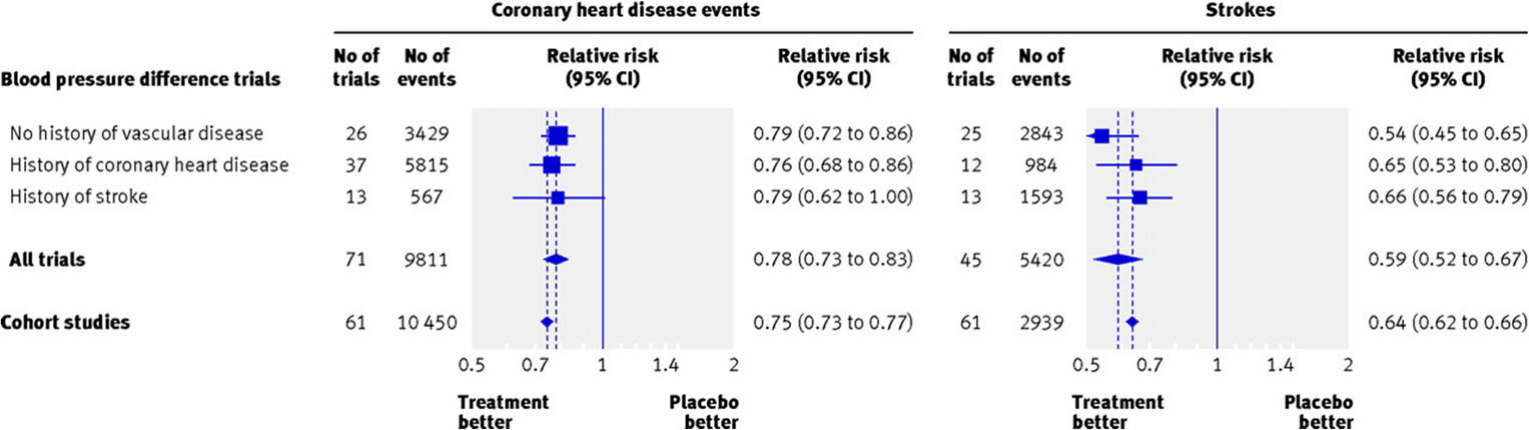
Relative risk for coronary heart disease and stroke in blood pressure difference trials, in epidemiological studies, and in the ACCORD trial. Reproduced, with permission, from reference [9]

Relative risk reduction for cardiovascular events in the ACCORD trial was within the predicted by the meta-analysis of risk [8] and confirmed by the meta-analysis of clinical trials [9]. There was a 13 % reduction in the incidence of coronary artery disease, in comparison with 22 % predicted by the meta-analysis of clinical trials (Fig. 1). The estimate of the meta-analysis was based in 71 studies with 9811 events, in comparison with 126 events of the ACCORD trial. For stroke, the relative risk reduction in the ACCORD trial was identical to that predicted by the meta-analysis of 45 studies, with 5420 events (Fig. 1). The size of benefit is remarkable, a relative risk reduction of 41 %. The absolute incidence of stroke in the ACCORD trial was unexpectedly low. Taking into account that the incidence of stroke is high worldwide and that it leads to devastating consequences, the decision to use 140 mmHg as the goal of therapy would be denying to patients with diabetes the benefit of preventing a large proportion of strokes.

## The Benefit of Treating Patients with Low Blood Pressure in Patients with Subclinical or Clinical Disease

There is a proof of the concept that high blood pressure is the major risk for cardiovascular disease [10•]. The size of benefit in clinical trials regarding the control of high blood pressure was within the estimations of risk provided by cohort studies. For a reduction of 10 mmHg in systolic or 5 mmHg of diastolic blood pressure, the relative risk reduction of coronary heart disease was 22 % (95 % CI from 27 to 17 %) in a meta-analysis of clinical trials, close to the estimation of reduction of 25 % (23 to 27 %) provided by a meta-analysis of cohort studies. The corresponding values for stroke were 41 % (33 to 48 %) in clinical trials compared to a cohort risk prediction of 36 % (34 to 38 %) [10•].

Randomized controlled trials done in patients with subclinical or clinical cardiovascular disease (heart failure, stroke, myocardial infarction, evidence of atherosclerosis, and diabetes) demonstrated significant reduction of cardiovascular events with the use of blood pressure-lowering agents independently of baseline blood pressure [11]. Table 1 presents the results of the more representative studies [12–19]. The benefit of treatment was mostly attributed to blood pressure-independent effects of the agents tested in these studies, the so-called pleiotropic effects. Nonetheless, the intensity of blood pressure reduction could explain by itself the benefits of treatment.

**Table 1:** Clinical trials showing the effectiveness of blood pressure-lowering drugs in the prevention of cardiovascular events in patients with normal blood pressure

Clinical condition	Studies [reference]	Active treatment	Primary outcome	RRR (95 % CI)
Diabetes mellitus^a^	MICRO-HOPE [12]	Ramipril	MI, stroke, or CV death	25 % (12 to 36)
Any evidence of atherosclerosis in the coronary, cerebral, or peripheral territories	HOPE [13]	Ramipril	MI, stroke, or CV death	22 % (14 to 30)^b^
	EUROPA [14]	Perindopril	MI, CV death, or cardiac arrest	20 % (9 to 29)^b^
Recovered from stroke	PROGRESS [15]	Indapamide plus perindopril	Stroke	42 % (19 to 58)
Asymptomatic heart failure	SOLVED [16]	Enalapril	CV deaths	12 % (−3 to 26)
Overt heart failure	SOLVED [17]	Enalapril	CV deaths	18 % (6 to 28)
	SAVE [18]	Captopril		21 % (5 to 35)
Class IV heart failure	CONSENSUS [19]	Enalapril	Total mortality	40 % (*P* = 0.002)

*RRR* relative risk reduction, *MI* myocardial infarction, *CV* cardiovascular

^a^In individuals at least 55 years old with another major cardiovascular risk factor (elevated cholesterol levels, low HDL cholesterol, cigarette smoking, or microalbuminuria)

^b^Estimate for the entire cohort, not significantly different between normotensive and hypertensive individuals

**Fig. 2:**
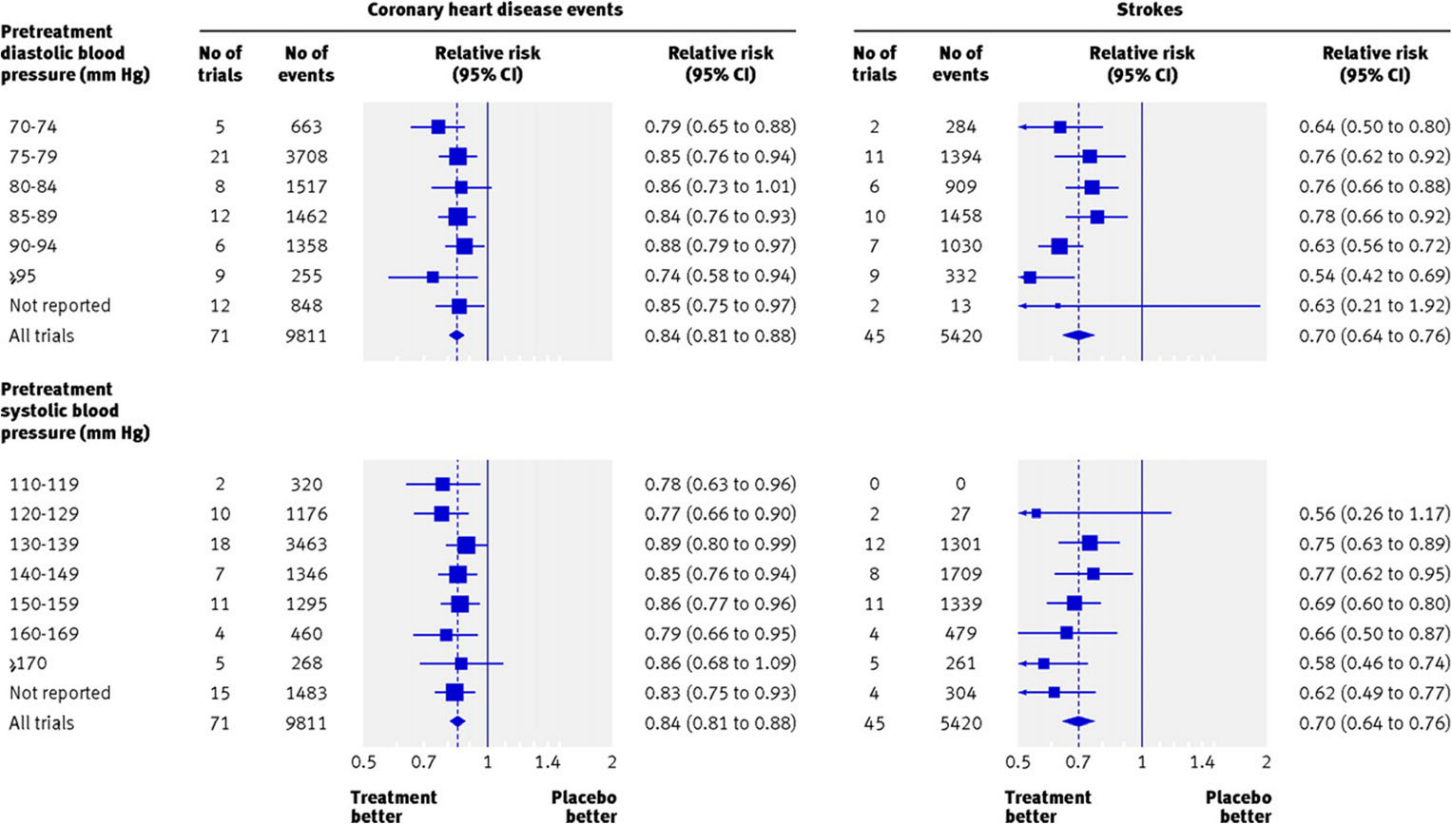
Relative risks for coronary events and stroke in patients stratified by blood pressure at the beginning of randomized controlled clinical trials. Reproduced, with permission, from reference [9]

The meta-analysis by Law and associates [9] demonstrated that the prevention of coronary artery disease and stroke with further reduction of blood pressure was independent of its values at the beginning of these trials (Fig. 2). Another meta-analysis of 25 RCT with patients with prehypertension and cardiovascular disease [19] demonstrated that participants receiving antihypertensive medication compared with controls had a mean reduction of 23 % in the incidence of stroke, 20 % in the incidence of myocardial infarction, 29 % for fatal and nonfatal heart failure, 15 % for composite cardiovascular events, 17 % for cardiovascular mortality, and 13 % for all-cause mortality. Thompson and coworkers [20] called for additional randomized trial to assess these outcomes in patients without cardiovascular disease. Nonetheless, these studies would be hardly feasible, in face of the very low absolute risk of patients with prehypertension free of cardiovascular disease. A large sample size and long time of follow-up would be required, and it would be difficult to get grants and persistence to doing such trial.

## Preventing Full Hypertension in Patients with Prehypertension

Besides the risks of prehypertension for cardiovascular disease, it is a precursor of hypertension in high proportion of individuals. Many studies have identified the cardiovascular risks of prehypertension and the incidence of hypertension [21–24]. In Porto Alegre, four in five individuals 40 to 49 years old with prehypertension would become hypertensive in 10 years [25].

Taken together, these evidences support the view that most guidelines moved to the wrong direction. Evidences still do not show the whole picture and should be completed by indirect evidences and analogous models to build the theory. If someone wants to live more than 100 years, he or she should keep blood pressure below 120 by 80 mmHg, which are the usual BP values of worldwide centenarians.

## The Efficacy of Drugs to Prevent Hypertension in Patients with Prehypertension

Two large clinical trials showed that the prevention of hypertension by drug treatment is feasible and well tolerated. In the TROPHY study [26], 772 individuals with systolic blood pressure between 130 and 139 mmHg or diastolic blood pressure between 85 and 89 mmHg were randomized to candesartan, 16 mg daily or placebo, besides recommendations to change lifestyle. After 2 years, the incidence of hypertension was 66 % lower in individuals treated with candesartan (relative risk 0.34, 95 % CI 0.25–0.44). After 2 years, the treatment was interrupted and blood pressure tended to return to the levels of the control group. The treatment was well tolerated.

The results of the TROPHY study originated an intense debate, mostly because of the criteria employed to diagnose hypertension. In the total, 70 % of the diagnoses were based on the detection of hypertensive levels at three different visits, not necessarily consecutive, which could have resulted from random transgressions of the prespecified thresholds on three occasions, rather than a change in the usual blood pressure of the patients [27]. According to Meltzer [28], the study showed a slow unmasking of hypertension and not the prevention of hypertension. The authors of the study TROPHY reanalyzed data according to the JNC 7 criteria for the definition of hypertension, confirming the original findings [29]. The authors of TROPHY trial have repeatedly stated that they do not advocate treatment of millions of people with prehypertension worldwide, recommending further studies.

In the study PHARAO [30], 1008 individuals with systolic blood pressure within the same values of the TROPHY trial were randomized to ramipril 5 mg daily or no treatment. Patients were followed-up for 3 years. The relative risk reduction for the incidence of hypertension was 34 % (hazard ratio 0.66; 95 % CI 0.53–0.81). Cough was more frequent in individuals treated with ramipril (4.8 vs. 0.4 %). The main limitation of this study was its open design, which could have increased the prescription of blood pressure-lowering drugs in the control group or decreased the frequency of prescription in the active treatment group. Prescription of antihypertensive agents was one of the criteria to diagnose incident hypertension.

There is no further study published looking at the effect of blood pressure-lowering drugs on the incidence of hypertension. Studies evaluated the effect of drug and nondrug interventions over BP in patients with prehypertension. The most interesting one compared the effect of aspirin 100 mg administered on awakening and at bedtime and with a group submitted to lifestyle change recommendations on 48-h BP measured by ambulatory blood pressure (ABP) monitoring [31]. There was a significant decreasing of BP on ABP monitoring among patients randomized to bedtime aspirin in comparison with the other two groups (approximately 7 and 4 mmHg for 24-h systolic and diastolic BP, respectively).

Nebivolol apparently reduced central BP compared to placebo in a randomized controlled trial. However, the authors did not test for the between-group differences and did not adjust for the higher baseline values of measurements of central hemodynamics in patients allocated to the active treatment [32]. A placebo-controlled randomized controlled trial showed no effect of aliskiren on the progression of coronary disease in patients with prehypertension [33].

An anti-stress therapy (mindfulness-based stress reduction) was compared with muscle relaxation training in a randomized controlled trial. There was a small reduction of office BP in the anti-stress therapy, but it was not confirmed by ABP monitoring [34]. In a small randomized trial, continuous positive airway pressure lowered BP in patients with severe obstructive sleep apnea and prehypertension or masked hypertension [35].

## Ongoing Trials

Studies examining the benefit of drug treatment of patients with prehypertension free of cardiovascular disease are warranted [20]. There are, however, few studies underway addressing this issue. A search in the US, European, and Chinese registers of clinical trials identified only two ongoing studies directly related to the clinical benefits of treating prehypertension. There are protocols for other studies in individuals with prehypertension, but none directly related to the prevention of hypertension, target organ damage, or clinical outcomes.

The CHINON study (http://www.chictr.org/en/proj/show.aspx?proj=554) aims to investigate whether low-dose antihypertensive treatment of with either indapamide, an angiotensin receptor blocker (ARB), reserpine compound, or placebo reduces the risk of cardiovascular events and development of hypertension and diabetes in high-normal blood pressure individuals with cardiovascular risk factors in China. In total, 10,806 subjects aged 45–79 years were randomized between 2008 and 2012. The primary endpoint is the combination of nonfatal stroke, nonfatal myocardial infarction, and fatal cardiovascular events. The secondary endpoints include the development of hypertension and diabetes. Details about the duration of the study were not reported.

We are conducting the PREVER study, a nationwide double-blind, placebo-controlled, randomized clinical trial in Brazil [36], which aims to explore the effectiveness of a low dose of an association of diuretics over the incidence of hypertension in individuals with prehypertension free of cardiovascular disease. The rationale is that diuretics act on the main mechanism of BP elevation with age in populations, antagonizing the loss of the kidney capacity to promote the excretion of sodium overload without increasing BP (pressure natriuresis) [37]. Moreover, it is expected that the response to the BP-lowering effect of diuretics may be higher before the development of structural abnormalities in large and resistant vessels and in the heart. The intervention aims to explore a window of opportunity to prevent the development of hypertension. The trial enrolled 724 participants who remained with prehypertension after 3 months of lifestyle recommendations. The active treatment is an association of chlorthalidone 12.5 mg with amiloride 2.5 mg. Patients have been followed-up for 18 months. The primary outcomes are the incidence of hypertension, adverse events, development of microalbuminuria, left ventricular hypertrophy in the EKG, and diabetes. Data collection finished in September 2014 and the results are expected to be reported in the following months.

## Conclusion

Guidelines, regulatory authorities, and researchers have been reluctant to recommend drug interventions for individuals with prehypertension. It is unlikely that in a foreseen future there will be definite evidences that drug intervention in individuals with prehypertension is the key to control the burden of hypertension. In the mean time, partial and indirect evidences may be used to support the idea that it is worth to interfere at this point of the natural history of hypertension. The results of the ongoing clinical trials may contribute to strengthen this interpretation.
